# Beyond the knife: HDR plesiotherapy brings precision to atypical fibroxanthoma

**DOI:** 10.3332/ecancer.2025.1968

**Published:** 2025-08-18

**Authors:** Carmen Saiz, Angel Montero, Mercedes López, Bruno Zambrana, Beatriz Alvarez, Jeannette Valero, Raquel Ciervide, Ovidio Hernando, Mariola Garcia-Aranda, Emilio Sanchez, Leyre Alonso, Xin Chen-Zhao, Rosa Alonso, Carmen Rubio

**Affiliations:** 1 Department of Radiation Oncology, HM Hospitales, Madrid, Spain; 2 Department of Medical Physics, HM Hospitales, Madrid, Spain

**Keywords:** atypical fibroxanthoma, plesiotherapy, customised mold

## Abstract

Background: Atypical fibroxanthoma is a rare cutaneous tumour of mesenchymal origin, often presenting as a rapidly growing, painless mass in sun-exposed areas. Although historically considered benign, it has an intermediate malignant potential with a risk of recurrence and, in rare cases, metastasis. Treatment primarily involves surgical excision, although recurrence rates can occur. Adjuvant superficial high-dose-rate (HDR) brachytherapy (plesiotherapy) is able to reduce recurrence risk, particularly after local tumour relapses and/or when complete excision with wide margins is unfeasible. It provides excellent local control, cosmetic outcomes and minimal toxicity, making it a promising option in selected cases. This report highlights the utility of customised-mold HDR plesiotherapy for a locally recurrent atypical fibroxanthoma.

## Introduction

Atypical fibroxanthoma (AFX) is a rare type of cutaneous tumour of mesenchymal origin composed of atypical spindle cells with benign behavior, first described in 1961 by Hewig [1], with a peak incidence in seventh-eighth decade of life. This tumour exhibits a wide range of morphological features, leading to variants such as spindle cell, clear cell, desmoplastic, granular, angiomatoid, hemosiderotic, myxoid and others [2]. For this reason, it is often referred to as a ‘great imitator’ of other tumours, making clinical diagnosis particularly challenging.

AFX typically presents as a rapidly growing painless mass, most commonly in sun-exposed areas and it is usually managed by wide local excision. However, contrary to initial beliefs, AFX is now recognised as a tumour with intermediate malignant potential harboring a non-negligible risk of relapse. A systematic retrospective review of the literature focusing on treatment modality found a recurrence and metastasis rate of 8.0% (5.6% when adjusted for incomplete excisions) and 0.5% for local excision and 4.6% and 3.2% for Mohs micrographic surgery (MMS), respectively [3]. Metastases are more commonly

observed in large tumours, those with necrosis, vascular invasion or after local recurrences, being more frequently observed among immunosuppressed patients. The most frequent metastatic sites include regional lymph nodes, the parotid gland (in cases of head tumours) and the lungs [2, 3].

In the absence of definitive clinical guidelines, we present a clinical case of a three-time relapsed AFX successfully managed by adding adjuvant superficial brachytherapy after new complete resection.

## Case Report

An 82-year-old male patient with no significant medical history consulted because of a painless growth of a nodular lesion located in the left frontoparietal region of the scalp. He reported daily sun exposure to his face and scalp (due to alopecia) for over 30 years without adequate protection (neither regular use of a hat nor sunscreen). A standard excisional procedure was performed. The pathological report macroscopically described a lesion of 43 × 5 × 21 mm. The histological and immunochemistry characteristics were consistent with Atypical fibroxanthoma diagnosis.

Two months after the first surgery, the patient consulted because of local regrowth. An incisional biopsy revealed a spindle-cell malignancy with clear surgical margins. Immunohistochemical analysis was negative for cytokeratin, CD34, actin and S100 but positive for KP1-PGM1, consistent with the diagnosis of AFX. The patient underwent wider excision with negative margins, confirming the diagnosis of AFX.

Three months later, a new 22 mm local recurrence was noted. A third surgical resection of AFX was performed with histologic confirmation, and the patient was subsequently referred to our Department of Radiation Oncology for consideration of adjuvant radiotherapy with the aim of reducing the risk of a new local recurrence.

After reviewing the medical records and the sequence of treatments received during follow-up, the patient was offered a superficial high-dose rate (HDR) brachytherapy (plesiotherapy).

A customised mold encompassing tumour bed on the left fronto-parietal area was made from eXaSkin^®^ (Anatomical Geometry S.L., Mairena del Aljarafe, Seville, Spain) bolus in paste form with 5 plastic tubes of 6Fr of diameter compatible with after-loading devices situated parallel and equidistant with a spacing of 12 mm between them. The number of tubes and the spacing between them were determined by the size of the treatment area, as defined by the radiation oncologist. The treatment field included a safety margin of at least 1 cm of uninvolved skin around the tumour or surgical bed to account for potential microscopic spread.

A computed tomography (CT) scan with 1-mm slice intervals was performed with the mold in place, containing wire dummies inserted into the plastic tubes. Prior to CT acquisition, the radiation oncologist meticulously delineated the skin area to be treated, corresponding to the surgical bed with 2 cm margin and marked it with a wire dummy. This clinical target volume (CTV) was delineated on each CT slice using Oncentra^®^ treatment planning software (Elekta AB, Stockholm, Sweden). No additional margin was added to create a planning target volume from the CTV.

**Figure 1.**
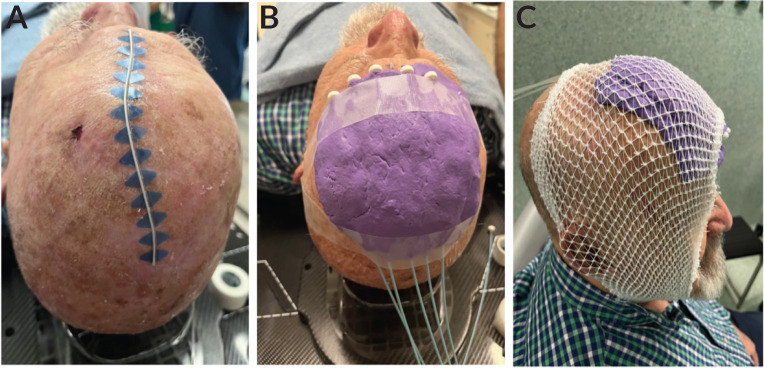
Customised mold with eXaSkin^®^ bolus paste and plastic tubes for superficial HDR plesyotherapy (A-C).

A total dose of 40 Gy was delivered in 10 daily fractions of 4 Gy each, administered Monday through Friday over two weeks. Treatment was performed using an after-loading HDR brachytherapy system equipped with a 192-Iridium radioactive source (MicroSelectron, Nucletron BV, Elekta AB, Stockholm, Sweden). Before each treatment fraction, the radiation oncologist carefully verified the precise repositioning of the mold to ensure complete coverage of the delineated skin area, thereby guaranteeing accurate dose delivery to the target. Figure 1 illustrates the treatment setup and radiotherapy dosimetry.

The plesiotherapy procedure was tolerated, with the patient experiencing moderate scalp erythema and localised Grade 2 acute skin toxicity, along with circumscribed areas of Grade 3 reaction 3 weeks after completing brachytherapy, which healed properly after local treatment. At the 6-month follow-up, the patient remained in good overall condition and continued to lead a normal life, with no signs of chronic toxicity. Physical examination revealed only flat surgical scars from previous resections, with no new lesions suspicious for malignancy detected.

## Discussion

AFX is a rapidly growing, exophytic tumour, usually painless and non-invasive, more frequent in men aged 70–80 and appearing on sun-exposed areas (head, face and neck in 80% of cases). Lesions are typically firm, under 2 cm, ulcerated or bleeding, with a reddish-brown hue. It is rare, accounting for 2% of scalp tumours and 0.002% of non-melanoma skin cancers in some studies [4, 5].

AFX consists of pleomorphic spindle cells with marked atypia. While lymphovascular and perineural invasion are rare, subcutaneous necrosis suggests increased aggressiveness. Immunohistochemically, AFX is positive for vimentin, CD10 and p53 and negative for markers distinguishing it from squamous cell carcinoma, melanoma and leiomyosarcoma. AFX shares histological features with undifferentiated pleomorphic sarcoma, although the latter is more aggressive, with higher recurrence (5%–28%) and metastasis rates and some early ‘metastatic AFX’ cases may have been misclassified as UPS [7].

There are no established guidelines for AFX treatment, which varies based on lesion invasiveness. Non-invasive cases can be managed with cryotherapy, laser, surgery or topical therapies, while invasive lesions require more definitive approaches. MMS is the preferred method, particularly in high-risk areas, as it allows intraoperative margin assessment, minimising tissue loss and recurrence [2]. Brown and Swanson [8] first advocated for MMS, reporting no recurrences. A meta-analysis reported recurrence rates of 2.0% with MMS and 8.7% with WLE, dropping to 5.6% when incomplete excisions were excluded. Recurrences occurred within 7.3–24 months (median: 31.5 months), while distant metastases (1%–4%) were more frequent in large, necrotic or invasive tumours with vascular involvement, typically appearing 24–28 months post-surgery [3]. Though AFX has low metastatic potential, its recurrence risk remains significant, prompting interest in alternative treatments. Chemotherapy has shown limited success, but emerging targeted therapies based on RAS mutations offer potential in advanced or unresectable cases. However, further research is needed before widespread clinical use [9, 10].

The use of radiotherapy (RT) for AFX has been rarely reported, mainly in metastatic cases or after multiple recurrences uncontrolled by surgery. Radiotherapy, traditionally employed as an adjuvant treatment post-surgery, is also regarded as a primary option when surgery is infeasible due to aesthetic considerations, comorbid conditions or tumour location. Various techniques, including external beam radiotherapy with either photons or electrons, have demonstrated high local control rates (87%–100% at 2 and 5 years) with excellent cosmetic outcomes and minimal toxicity. Typically, large lesions are treated with electron-based radiation using a linear accelerator, whereas smaller lesions are managed with photons through kilovoltage units or plesiotherapy [9, 10]. Brachytherapy was initially explored in the 1970s due to high recurrence rates, but detailed information on RT characteristics remained scarce until Dogget et al. [12] provided a comprehensive report on electronic brachytherapy (50 keV, 40 Gy in 2 weekly fractions), achieving a local recurrence rate of 12.5% over 23.7 months [11]. Currently, RT is considered an adjuvant option when surgery is insufficient or contraindicated, particularly in high-risk cases. Brachytherapy achieves similar outcomes with excellent cosmetic results and minimal toxicity, and the use of customised molds, frequently used for superficial brachytherapy – plesiotherapy – have evolved with 3D printing technology, enhancing precision and adaptability to complex anatomical regions [12]. In our case, we utilised a two-layer mold with 6Fr catheters, placed in a parallel and equidistant manner, covering the entire surgical bed with a safety margin. CT imaging (1-mm slices) was used for precise catheter reconstruction and target delineation. Following international guidelines, we selected a regimen of 40 Gy in 10 daily fractions, ensuring good tolerance and efficacy [13, 14]. This approach highlights the importance of advanced imaging and individualised mold fabrication in optimising brachytherapy for non-melanoma skin tumours.

In conclusion, HDR plesiotherapy appears to be a valuable option in the multidisciplinary management of atypical fibroxanthoma and may serve as an effective treatment approach in patients with tumour recurrence following initially curative surgery. HDR plesiotherapy using a personalised mold facilitates the treatment of irregular or hard-to-access areas, ensuring adequate dosimetric coverage while protecting the surrounding healthy tissues. This approach has been shown to be feasible for local management of AFX, associated with good clinical and cosmetic outcomes, with excellent patient tolerance.

## Conflicts of interest

All the authors declare no conflict of interest regarding any aspect of this work.

## Funding

The authors declare that there is no source of funding for this work.

## Figures and Tables

**Figure 2.**
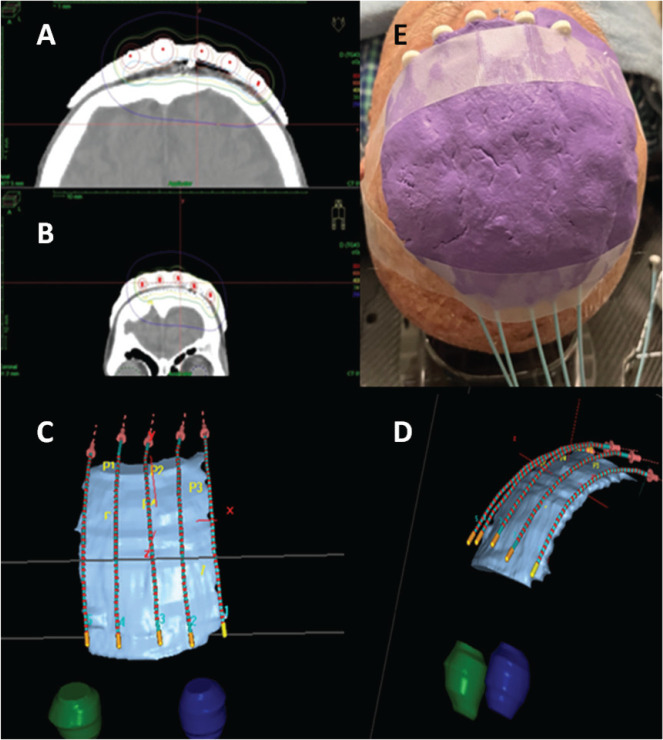
HDR plesyotherapy planning showing isodose distributions and 3D mold with plastic catheters reconstruction (A-E).
